# Close association between non-alcoholic fatty liver disease and ossification of the posterior longitudinal ligament of the spine

**DOI:** 10.1038/s41598-021-96714-9

**Published:** 2021-08-31

**Authors:** Tsutomu Endo, Yoshinao Koike, Hideaki Miyoshi, Yuichiro Hisada, Ryo Fujita, Ryota Suzuki, Masaru Tanaka, Takeru Tsujimoto, Yukitoshi Shimamura, Yuichi Hasegawa, Masahiro Kanayama, Tomoyuki Hashimoto, Fumihiro Oha, Naoki Noro, Kiyofumi Komano, Misaki Ishii, Yoichi M. Ito, Norimasa Iwasaki, Masahiko Takahata

**Affiliations:** 1 Department of Orthopedic Surgery, Hokkaido University Graduate School of Medicine, Kita-15 Nishi-7, Kita-ku, Sapporo, Hokkaido 060-8638 Japan; 2 Department of Orthopedics, Hakodate Central General Hospital, 33-2 Hon-cho, Hakodate, Hokkaido 040-8585 Japan; 3 Division of Diabetes & Obesity, Faculty of Medicine & Graduate School of Medicine, Hokkaido University, Kita-15 Nishi-7, Kita-ku, Sapporo, Hokkaido 060-8638 Japan; 4 Clinical Research and Medical Innovation Center, Hokkaido University Hospital, Kita-14 Nishi-5, Kita-ku, Sapporo, Hokkaido 060-8648 Japan

**Keywords:** Spine structure, Risk factors, Metabolic disorders

## Abstract

Ossification of the posterior longitudinal ligament (OPLL) of the spine is a disease of unknown etiology occurring frequently in individuals with metabolic disturbances. Obesity has been suggested as a potential risk factor for the severity of OPLL. We aimed to investigate whether non-alcoholic fatty liver disease (NAFLD) is associated with OPLL severity. We assessed the severity of NAFLD by a liver-to-spleen (L/S) ratio on computed tomography (CT) scans of 85 symptomatic OPLL patients at a single institution in Japan. We also assessed the severity of OPLL by CT reconstruction sagittal and axial images. The prevalence of NAFLD in middle-aged patients (age < 70 years, n = 50) was 80.3%, which was 2.5–8 times higher than that in the general Japanese population (9–30%). The ossification index of the spinal ligaments increased in proportion to the severity of fatty liver. The L/S ratio was revealed as a significant risk factor associated with the total ossification index (standardized β: -0.40, 95% confidence interval − 54.34 to − 4.22). This study suggests the potential contribution of NAFLD to the progression of OPLL. The close association between NAFLD and OPLL demonstrated in this study warrants further study to elucidate the causal nature of this relationship.

## Introduction

Heterotopic ossification of the posterior longitudinal ligament (OPLL) is a disease of unknown etiology that most frequently affects the cervical spine, causing myelopathy^1–4^. However, recent studies have demonstrated that there is a clinically important patient subgroup that develops OPLL in other regions, including the thoracic and lumbar spine^5–7^. Epidemiological studies have revealed that patients with thoracic OPLL have a strong tendency toward an early onset of symptoms and a high prevalence of morbid obesity, as well as co-existing ossified lesions of spinal ligaments over the entire spine^8,9^. This prompted us to further investigate the association between obesity-related diseases and OPLL.

Non-alcoholic fatty liver disease (NAFLD) is very common among individuals with obesity, and it is attributable to over-nutrition, a sedentary lifestyle, sarcopenia, and genetic background^10,11^. NAFLD is also closely related to metabolic disturbances. Therefore, there is an on-going international effort to rename NAFLD to metabolic dysfunction-associated fatty liver disease (MAFLD)^12^. MAFLD is diagnosed by evidence of hepatic steatosis as well as concomitant diabetes mellitus type 2, being overweight, or having metabolic disturbances, all of which are frequently observed in patients with OPLL^10–16^. Despite the shared features between NAFLD and OPLL, no previous studies have explored the association between them. This may be due to inadequate awareness of the presence of NAFLD by physicians, as well as the patients themselves, as most patients with NAFLD remain asymptomatic and liver function tests mostly show normal results, with mild elevations in aminotransferases^17–19^. Thus, it is of great interest to determine whether patients with OPLL have a high prevalence of NAFLD, as well as whether there is a link between NAFLD and OPLL.

At present, there is no therapeutic approach based on the progression of OPLL, and surgery is the only treatment option used to relieve myelopathy. In order to develop novel therapeutic strategies against OPLL, it is necessary to explore the underlying etiology associated with severe OPLL. Therefore, we aimed to determine the prevalence of NAFLD in symptomatic patients with OPLL and to assess whether the severity of fatty liver is associated with the severity of OPLL.

## Methods

### Study design

A retrospective cross-sectional study was conducted. All patients were included between June 2019 and May 2020. The study was conducted in accordance with the Declaration of Helsinki (1964) and was approved by the ethics committee of the Hakodate Central Hospital and Hokkaido University Hospital. Written informed consent was obtained from all participants.

### Patients

All patients with OPLL were diagnosed and followed up by a spine surgeon or radiologist based on clinical symptoms and computed tomography (CT) images at the time of diagnosis. Of the 94 patients who regularly visited a single institution, 6 patients who had not undergone CT scans including liver, spleen, and entire spine were excluded. Three patients who regularly consumed alcohol (30 g/day or more for men and 20 g/day or more for women) and were at risk of developing alcoholic liver disease were excluded^18,19^. Finally, a total of 85 patients (35 males and 50 females) were included in this study.

### Grouping of patients with OPLL according to their age and OPLL type

In the evaluation of the association between fatty liver and OPLL, patients were divided into middle-aged (age < 70 years, n = 50) and elderly groups (age ≥ 70 years, n = 35). This grouping was performed because fat deposits in the hepatocytes tend to disappear as liver fibrosis progresses from the eighth decade of life onward (> 70 years)^20–22^, and OPLL is presumed to develop in the sixth (50–60 years old) and seventh (60–70 years old) decade of life, or earlier^23–25^.

We also divided patients according to OPLL type: patients with OPLL only in the cervical spine were classified into the localized-OPLL group, and those with OPLL in the thoracolumbar spine, with or without cervical spine involvement, were classified into the diffuse-OPLL group. This classification was based on our previous study showing that patients with myelopathy caused by thoracic OPLL have distinct features, such as severe obesity, early symptom onset, and diffuse ossification of the spinal ligaments^8,9^.

### Demographics, comorbidities, and OPLL distribution

Demographic data were obtained from all participants. Their current body mass index (BMI), comorbidities (diabetes, hypertension, hyperlipidemia, heart disease, kidney disease, hyperuricemia, and cancer), and drinking habits were assessed. ​The distribution of OPLL was assessed by sagittal reconstruction computed tomography (CT) images of the entire spine.

### Image acquisition

Non-contrast CT scans involving the liver, spleen, and entire spine were obtained for each patient using a CT-Aquilion ONE™ / GENESIS Edition system (Canon Medical Systems Corporation, Tochigi, Japan). To reduce motion artifacts, three consecutive scans were performed while the patient held their breath.

### BMI criteria by World Health Organization (WHO) and the Japan Society for the Study of Obesity (JASSO) guidelines

For adults, the current WHO guidelines define BMI: 18.5–24.9 kg/m^2^ as the normal range, BMI ≥ 25 kg/m^2^ as overweight, BMI ≥ 30 kg/m^2^ as obese, and BMI ≥ 40 kg/m^2^ as highly obese^26^. In 2002, the WHO Expert Committee proposed to lower the BMI cut-off point, which would trigger public health action among Asian people, categorizing BMI: 18.5–22.9 kg/m^2^ as the normal range, BMI: 23–27.4 kg/m^2^ as overweight, and BMI ≥ 27.5 kg/m^2^ as obese^26,27^. The JASSO guidelines set BMI cut-off points of 25 kg/m^2^ as obese and 35 kg/m^2^ as highly obese^28^. JASSO defines BMI: 18.5–24.9 kg/m^2^ as the normal range, BMI: 25.0–29.9 kg/m^2^ as obese class I, BMI: 30.0–34.9 kg/m^2^ as obese class II, BMI: 35.0–39.9 kg/m^2^ as obese class III, and BMI ≥ 40.0 kg/m^2^ as obese class IV.

### Evaluation of liver fat

Liver fat measurements were performed independently by two radiology technologists (NN and KK) using Ziostation2 imaging software (Ziosoft, Tokyo, Japan), and the mean value was adopted. Following the guidelines of the Japanese Society of Gastroenterology^29,30^, the attenuation values (Hounsfield units; HU) of the liver and spleen were measured using regions of interest (ROIs) with an area of 400 mm^2^. ​Two ROIs in the right lobe of the liver, two in the left lobe, and two in the spleen were used as the largest possible areas. ​Care was taken to exclude hepatic vessels and artifacts, and all measurements were obtained in areas with uniform parenchymal attenuation. HU values measured in each segment of the liver and spleen were averaged. The liver-to-spleen HU ratio (L/S ratio) was calculated as the mean attenuation value of the liver and spleen. In this study, an L/S ratio ≤ 1.0 on CT indicated severe fatty liver, 1.0 < L/S ratio < 1.2 indicated mild fatty liver, and an L/S ratio ≥ 1.2 indicated non-fatty liver. The percentage of estimated intrahepatic fat deposition was based on the cut-off values of the guidelines of The Japanese Society of Gastroenterology: L/S ratio 0.9: ≥ 60%, L/S ratio 1.01: 30–60%, L/S ratio 1.12: ≤ 30%, L/S ratio 1.2: no fat deposition^29^.

### Liver fibrosis index based on laboratory tests and anthropometric parameters

The fibrosis index based on four factors (FIB-4) was calculated using the following equation: FIB-4 = age (years) × aspartate aminotransferase (AST; U/L) / [platelet count (10^9^ /L) × √ alanine aminotransferase (ALT; U/L)] ^31^. Patients who scored < 1.3 and > 2.67 on the FIB-4 index are considered to be at low and high risks of advanced fibrosis, respectively^32,33^. With increasing age, the false-positive rate of liver fibrosis increases, leading to a decrease in the ability to diagnose based on the FIB-4 index^33^. We referred to the recently proposed cut-off points of the FIB-4 index appropriate for each age group in the Japanese population; a low cut-off point and a high cut-off point, respectively: 1.05 and 1.21 for ≤ 49 years, 1.24 and 1.96 for 50–59 years, 1.88 and 2.67 for 60–69 years, and 1.95 and 2.67 for ≥ 70 years^21^. The proportion of patients whose scores were above or below the cut-off point in this study was evaluated for each age group and then combined.

### Severity of spinal ligament ossification

The distribution of spinal ligament ossification (i.e., cervical, thoracic, and/or lumbar), including OPLL, ossification of the anterior longitudinal ligament (OALL), ossification of the ligamentum flavum (OLF), and ossification of the supra/interspinous ligaments (OSIL), was evaluated using sagittal reconstruction CT images of the entire spine. To determine the severity of spinal ligament ossification, the OPLL-index, OALL-index, OLF-index, OSIL-index, and total ossification index (defined as the sum of the presence or absence of ossification at each vertebral and intervertebral level) were calculated according to a previously described method^5,9^. The analysis was performed independently by three board-certified spine surgeons (TE, MT, and YH). Before reviewing the images, all readers interpreted the same images for 20 patients and an interobserver agreement was determined. The intraclass correlation coefficient (ICC) among the three observers was 0.96, and the 95% confidence interval (CI) was 0.92–0.98, indicating an extremely high interobserver agreement.

### Statistical analysis

Data were analyzed using BellCurve for Excel software (version 3.10; Social Survey Research Information Co., Ltd., Tokyo, Japan). Statistical significance was set at *P* < 0.05. Normality of data was tested using the Shapiro–Wilk test. The differences in continuous variables between the two groups were evaluated with the Student's t-test (for normally distributed data) or the Mann–Whitney U-test (for non-normally distributed data). Results are presented as mean value ± standard deviation for parametric variables (normally distributed data) or median (minimum, maximum) for non-parametric variables (non-normally distributed data). The difference in proportions between the two groups were evaluated with Fisher's exact test. Three-group comparisons were evaluated using the Kruskal–Wallis test. The relationship between the independent factors and the severity of spinal ligamentous ossification was analyzed by multiple regression analysis. The ICC was analyzed using a two-way mixed-effect model.

## Results

### Prevalence of fatty liver in middle-aged patients with OPLL and the distinct clinical features of patients with OPLL with severe fatty liver

Most patients with severe myelopathy had a history of spinal surgery. There were no patients with acute viral hepatitis, autoimmune hepatitis, primary biliary cirrhosis, cholelithiasis, or obstructive jaundice. None of the patients were using medications that cause hepatic steatosis, such as prednisolone or methotrexate. There were no patients with low nutritional status, including patients with gastrointestinal diseases such as inflammatory bowel disease, or cancer patients who had difficulty eating. The prevalence of fatty liver was 80.3% among all middle-aged patients with OPLL. Of the 50 middle-aged patients with OPLL, 68.1% had obesity (> 27.5 kg/m^2^) according to the WHO Asian BMI criteria^26,27^. The prevalence of fatty liver in patients with obesity was 93.3%, which was significantly greater than that in patients without obesity (64.2%) (*P* = 0.013).

Differences in the clinical characteristics of middle-aged patients with OPLL in the non-fatty liver, mild fatty liver, and severe fatty liver groups are shown in Table 1. Compared to those in the non-fatty liver group, patients in the severe fatty liver group had a significantly younger symptom onset age (*P* = 0.013) and significantly higher current BMI value (*P* = 0.023). There was also a tendency, albeit insignificant, toward a younger symptom onset age and higher current BMI value in the mild fatty liver group than in the non-fatty liver group. The proportions of comorbidities were similar among the three groups; however, the prevalence of hypertension, hyperlipidemia, and diabetes mellitus was higher in the three groups than in the general Japanese population^34^.

**Table 1:** Comparison of clinical characteristics and OPLL types among middle-aged patients (age < 70 years) with non-fatty, mild fatty, and severe fatty liver.

Variable	Non-fatty liver (n = 9)	Fatty liver	
		Mild fatty liver (n = 18)	Severe fatty liver (n = 23)
Current age (years)	64 (50–69)	58 (34–69)	59 (43–69)
Age of OPLL symptom onset (years)	58 (50–69)	54 (29–66)	50 (41–65)
Age of onset < 50 years (%)	0	33.3	47.8
Male (%)	44.4	38.8	31.2
Current BMI (kg/m^2^)	25.6 (23.5–31.8)	28.6 (23.4–37.2)	31.7 (21.0–39.1)
Current BMI ≥ 25.0 kg/m^2^ (%)	57.1	88.2	90.9
Current BMI ≥ 27.5 kg/m^2^ (%)	28.1	66.6	73.9
Current BMI ≥ 30.0 kg/m^2^ (%)	14.2	35.2	59.1
Current BMI ≥ 35.0 kg/m^2^ (%)	0	11.7	22.7
Platelet (10^9^ /L)	275 (148–274)	229 (140–383)	246 (104–437)
AST (U/L)	32.7 ± 18.7	25.8 ± 15.6	28.1 ± 11.1
ALT (U/L)	38 (12–72)	19 (11–59)	30 (13–64)
AST/ALT	0.9 (0.5–1.2)	1.1 (0.5–1.7)	0.8 (0.6–1.9)
**Comorbidity (%)**			
Myocardial infarction	0	0	4.3
Angina pectoris	11.1	5.5	8.7
Hypertension	55.5	38.8	69.5
Hyperlipidemia	33.3	50.0	34.7
Diabetes mellitus	11.1	44.4	43.4
Cancer	0	5.5	4.7
Renal disease	0	11.1	0
Gout	0	16.6	8.6
Drinking alcohol habits (%)	22.2	27.7	17.4
**Types of OPLL (%)**			
Localized-OPLL	55.5	11.1	17.3
Diffuse-OPLL	11.1	88.9	82.6

Data are shown as mean ± standard deviation for normally distributed variables and median (minimum–maximum) for non-normally distributed variables, and as the percentage. Patients were divided into three groups according to the liver-to-spleen ratio (L/S ratio) on computed tomography. The non-fatty liver group includes patients with an L/S ratio ≥ 1.2; the mild fatty liver group includes patients with 1.0 < L/S ratio < 1.2; and the severe fatty liver group included patients with an L/S ratio ≤ 1.0.

OPLL, ossification of the posterior longitudinal ligament; BMI, body mass index; L/S liver-to-spleen.

### Higher prevalence of fatty liver in diffuse-OPLL than in the localized-OPLL group among middle-aged patients with OPLL

**Figure 1:**
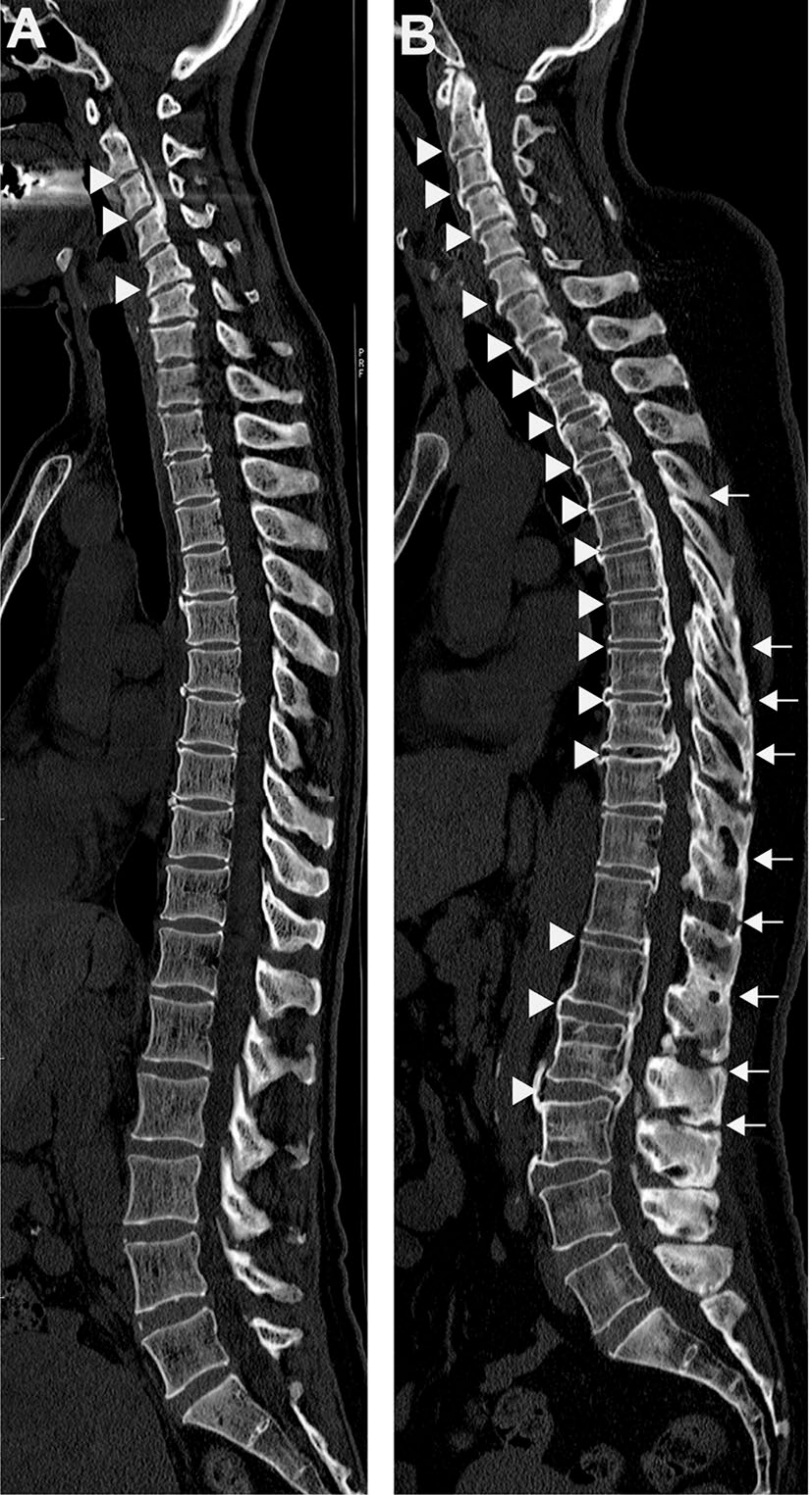
Sagittal reconstruction CT images of the entire spine in patients with OPLL. (**A**) A representative case from the localized-OPLL group. A 57-year-old woman without NAFLD (the L/S ratio: 1.41) had OPLL (arrow heads) in the cervical spine. (**B**) A representative case from the diffuse-OPLL group. A 49-year-old woman with NAFLD (the L/S ratio: 0.94) had multilevel OPLL and OALL of the whole spine (arrow heads) and multilevel OLF and OSIL in the thoracolumbar spine (arrows). NAFLD, non-alcoholic fatty liver disease; OPLL, ossification of the posterior longitudinal ligament; OALL, ossification of the anterior longitudinal ligament; OLF, ossification of the ligamentum flavum; OSIL, ossification of the supra/interspinous ligaments.

Since the proportion of patients with diffuse-OPLL was much higher in the fatty liver group than in the non-fatty liver group (Table 1), we compared the degree of liver fat and liver fibrosis between the localized-OPLL and diffuse-OPLL groups (Fig. 1 and Table 2). Although there were no statistically significant differences in the L/S ratio between the localized-OPLL and diffuse-OPLL groups, the proportion of patients with fatty liver (the L/S ratio < 1.2) was significantly higher in the diffuse-OPLL group than in the localized-OPLL group (*P* = 0.004). The FIB-4 index and the proportion of patients with low and high risks of liver fibrosis were not statistically significantly different between the localized-OPLL and diffuse-OPLL groups. Regarding background variables, the proportions of female patients and patients with diabetes mellitus were significantly higher in the diffuse-OPLL group than in the localized-OPLL group. The proportions of patients with BMI > 25 kg/m^2^ and BMI > 27.5 kg/m^2^ were comparable between the two groups. The proportions of patients with BMI > 30 kg/m^2^ and BMI > 35 kg/m^2^ were higher in the diffuse-OPLL group than in the localized-OPLL group, but the difference was not statistically significant.

**Table 2:** Comparison of the severity of fatty liver and liver fibrosis between middle-aged patients with localized-OPLL and diffuse-OPLL.

Variable	Localized-OPLL (n = 11)	Diffuse-OPLL (n = 37)	P-value
Current age (years)	61 (50–69)	60 (34–69)	0.825
Age of OPLL symptom onset (years)	54 (47–68)	51 (29–68)	0.268
Male (%)	81.2	35.1	0.013
Current BMI (kg/m^2^)	28.6 (24.0–31.8)	29.7 (21.0–39.1)	0.205
Current BMI ≥ 25.0 kg/m^2^ (%)	88.8	85.7	0.643
Current BMI ≥ 27.5 kg/m^2^ (%)	66.6	68.5	0.463
Current BMI ≥ 30.0 kg/m^2^ (%)	33.3	48.5	0.477
Current BMI ≥ 35.0 kg/m^2^ (%)	0	20.0	0.313
Platelet (10^9^ /L)	249 (196–363)	239 (104–437)	0.822
AST (U/L)	25 (14–33)	24 (12–68)	0.869
ALT (U/L)	34 (11–64)	25 (11–64)	0.323
AST/ALT	0.8 (0.5–1.2)	0.9 (0.6–1.9)	0.088
**Comorbidity (%)**			
Hypertension	45.4	56.7	0.731
Hyperlipidemia	27.2	40.5	0.499
Diabetes mellitus	9.0	45.9	0.035
**Fatty liver**			
L/S ratio	1.08 (0.64–1.40)	0.97 (0.44–1.48)	0.158
L/S ratio < 1.2 (%)	54.5	94.6	0.004
L/S ratio ≤ 1.0 (%)	36.3	51.3	0.498
**Liver fibrosis**			
FIB-4 index	1.05 (0.76–1.68)	1.15 (0.46–4.93)	0.611
FIB-4 index < 1.3 (%)	77.7	64.7	0.693
FIB-4 index < low cutoff point (%) ^a^	100	67.6	0.083
FIB-4 index > 2.67 (%)	0	5.7	1.000
FIB-4 index > high cutoff point (%) ^b^	0	11.7	0.563

Data are shown as median (minimum–maximum) and as the percentage. Patients were divided into two groups according to the OPLL type. Fatty liver was defined as an L/S ratio < 1.2.　Severe fatty liver was defined as an L/S ratio ≤ 1.0. A low risk of advanced liver fibrosis was defined as an FIB-4 index < 1.3. A high risk of advanced liver fibrosis was defined as an FIB-4 index > 2.67.

a. low cut-off points are 1.05 for ≤ 49 years, 1.24 for 50–59 years, 1.88 for 60–69 years, and 1.95 for ≥ 70 years, respectively.

b. high cut-off points are 1.21 for ≤ 49 years, 1.96 for 50–59 years, 2.67 for 60–69 years, and 2.67 for ≥ 70 years, respectively.

OPLL, ossification of the posterior longitudinal ligament; BMI, body mass index; L/S, liver-to-spleen; FIB-4, fibrosis index based on four factors.

### Association between fatty liver and heterotopic spinal ligament ossification among middle-aged patients with OPLL

**Figure 2:**
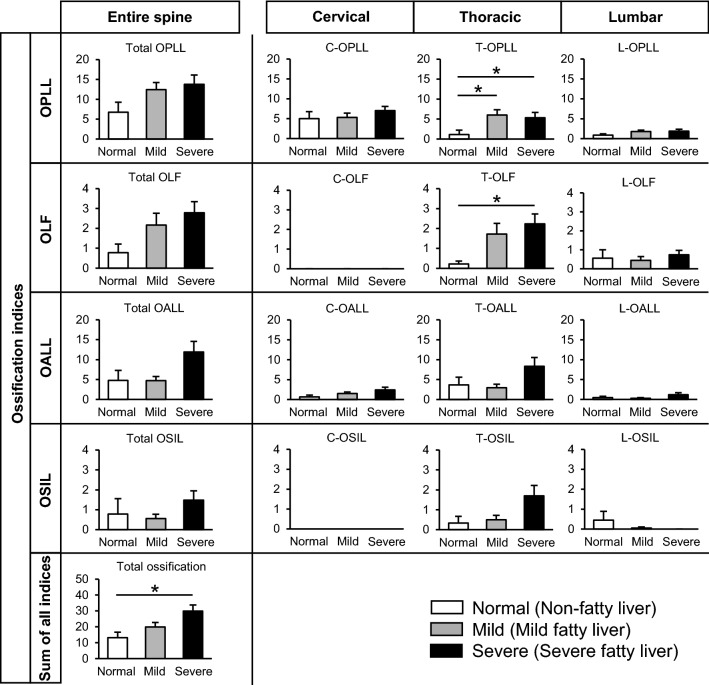
Comparison of the severity of ossification of the spinal ligaments among middle-aged patients with non-fatty liver, mild fatty liver, and severe fatty liver. Bars and error bars indicate the mean and the standard error of the mean, respectively. *: *P* < 0.05. C: cervical, T: thoracic, L: lumbar.

The modified ossification index was used to compare the severity of spinal ligament ossification among the non-fatty, mild fatty, and severe fatty liver groups (Fig. 2). The OPLL index in the thoracic spine (T-OPLL index), OLF index in the thoracic spine (T-OLF index), and the sum of all ossification indices were significantly higher in the severe fatty liver group than in the non-fatty liver group. The T-OPLL index was also significantly higher in the mild fatty liver group than in the non-fatty liver group. Notably, the sum of all ossification indices increased in proportion to the severity of fatty liver.

In a single regression analysis to examine factors associated with the sum of all ossification indices, the L/S ratio was identified as the sole risk factor (*P* = 0.008). Multiple regression analysis using the FIB-4 index and the L/S ratio as well as the previously reported risk factors such as age of symptom onset, BMI, and diabetes mellitus as independent variables revealed that the L/S ratio was associated with the sum of all ossification indices (*P* = 0.023) (Table 3) ^8,9,13–15^.

**Table 3:** Multiple regression analysis for factors associated with the sum of all ossification indices.

Independent variables	Univariate		Multivariate		
	β (95% CI)	*P* value	β	Standardized β (95% CI)	*P* value
Age (years)	0.07 (− 0.45 to 0.60)	0.779			
Age of symptom onset (years)	− 0.19 (− 0.71 to 0.32)	0.453	− 0.11	− 0.06 (− 0.66 to 0.42)	0.668
Sex	2.94 (− 6.47 to 12.36)	0.523			
BMI (kg/m^2^)	0.61 (− 0.51 to 1.75)	0.278	− 0.29	− 0.07 (− 1.64 to 1.05)	0.659
Hypertension	5.74 (− 3.56 to 15.04)	0.221			
Hyperlipidemia	3.06 (− 6.47 to 12.60)	0.521			
Diabetes mellitus	5.52 (− 4.01 to 15.05)	0.250	3.19	0.09 (− 7.73 to 14.13)	0.557
FIB-4 index	1.78 (− 3.16 to 6.72)	0.471	2.21	0.13 (− 2.92 to 7.34)	0.388
L/S ratio	− 23.98 (− 41.63 to − 6.32)	0.008	− 29.28	− 0.40 (− 54.34 to − 4.22)	0.023

β, regression coefficient; CI, confidence interval; OPLL, ossification of the posterior longitudinal ligament; BMI, body mass index; FIB-4, fibrosis index based on four factors; L/S; liver-to-spleen.

### Association between OPLL and liver fibrosis in elderly patients with OPLL

We analyzed the L/S ratio and the FIB-4 index to evaluate whether elderly patients in the diffuse-OPLL group had a higher chance of developing liver fibrosis due to fatty liver in middle age, compared with those in the localized-OPLL group. The clinical characteristics of the elderly patients with OPLL are shown in Table 4. There were no significant differences in the current age and prevalence of comorbidities between the two groups. The overall prevalence of fatty liver was 42.8% among elderly patients with OPLL, with no statistically significant difference in the L/S ratio between the two groups (Table 5). The proportions of patients with the FIB-4 index below the low cut-off point (< 1.95) and above the high cut-off point (> 2.67) in all elderly patients with OPLL were 57.1% and 11.4%, respectively. As expected, the diffuse-OPLL group had a significantly higher mean FIB-4 index than the localized-OPLL group (*P* = 0.048) (Table 5). The proportion of patients at a low risk of advanced liver fibrosis in the diffuse-OPLL group was significantly lower than that in the localized-OPLL group (*P* = 0.027). The proportion of patients at a high risk of advanced liver fibrosis in the diffuse-OPLL group was higher than that in the localized-OPLL group, but the difference was not statistically significant (Table 5).

**Table 4:** Comparison of clinical characteristics between elderly patients with localized-OPLL and diffuse-OPLL (age ≥ 70 years).

Variable	Localized-OPLL (n = 10)	Diffuse-OPLL (n = 25)
Current age (years)	75 (73–88)	80 (70–86)
Age of OPLL symptom onset (years)	65 (50–80)	70 (20–80)
Age of onset < 50 years (%)	10.0	16.0
Male (%)	90.0	16.7
Current BMI (kg/m^2^)	26.3 (19.1–31.2)	25.4 (15.6–34.7)
Current BMI ≥ 25.0 kg/m^2^ (%)	75.0	60.0
Current BMI ≥ 27.5 kg/m^2^ (%)	37.5	24.0
Current BMI ≥ 30.0 kg/m^2^ (%)	25.0	12.0
Current BMI ≥ 35.0 kg/m^2^ (%)	0	0
Platelet (10^9^ /L)	218 (159–309)	215 (126–362)
AST (U/L)	20 (19–33)	19 (12–48)
ALT (U/L)	26.4 ± 17.0	15.6 ± 10.7
AST/ALT	0.9 (0.4–1.6)	1.4 (0.8–3.6)
**Comorbidity (%)**		
Myocardial infarction	10.0	0
Angina pectoris	0	8.0
Hypertension	50.0	68.0
Hyperlipidemia	50.0	48.0
Diabetes mellitus	20.0	36.0
Cancer	20.0	20.0
Renal disease	0	0
Gout	0	12.0
Drinking alcohol habits (%)	30.0	20.0

Data are shown as mean ± standard deviation for normally distributed variables and median (minimum–maximum) for non-normally distributed variables, and as the percentage. The patients were divided into two groups according to OPLL type.

OPLL, ossification of the posterior longitudinal ligament; BMI, body mass index.

**Table 5:** Comparison of the severity of fatty liver and liver fibrosis between elderly patients with localized-OPLL and diffuse-OPLL.

Variable	Localized-OPLL (n = 10)	Diffuse-OPLL (n = 25)	P-value
L/S ratio	1.16 (0.77–1.60)	1.22 (0.37–1.67)	0.627
L/S ratio < 1.2 (%)	50.0	40.0	0.711
L/S ratio ≤ 1.0 (%)	30.0	12.0	0.321
FIB-4 index	1.67 ± 0.37	2.29 ± 1.08	0.048
FIB-4 index < 1.95 (%)	80.0	44.0	0.027
FIB-4 index > 2.67 (%)	0	20.0	1.000

Data are shown as mean ± standard deviation for normally distributed variables and median (minimum–maximum) for non-normally distributed variables, and as the percentage. Patients were divided into two groups according to the OPLL type. Fatty liver was defined as an L/S ratio < 1.2. Severe fatty liver was defined as an L/S ratio ≤ 1.0. A low risk of advanced liver fibrosis was defined as an FIB-4 index < 1.95. A high risk of advanced liver fibrosis was defined as an FIB-4 index > 2.67.

OPLL, ossification of the posterior longitudinal ligament; L/S, liver-to-spleen; FIB-4, fibrosis index based on four factors.

## Discussion

The current study revealed that the prevalence of NAFLD in symptomatic patients with OPLL aged 70 years or younger was surprisingly high at approximately 80%, which was 2.5–8 times higher than that in the general Japanese population (9–30%)^20,22^. The clinical significance of this finding is that most patients with OPLL with identified fatty liver on CT had not been diagnosed with NAFLD, which may be attributable to the unique nature of the disease, as most patients with NAFLD remain asymptomatic, and the liver function tests are mostly normal or show mild elevations in aminotransferases until the liver fibrosis progresses^17–19^. A possible explanation for the high prevalence of NAFLD in symptomatic patients with OPLL is the multiple risk factors for NAFLD, such as obesity, physical inactivity, a sedentary lifestyle, and sarcopenia, which are accompanying symptoms of myelopathy^8,9,13–16^.

Our results suggest that fatty liver is a contributing factor in the progression of spinal ligament ossification. This was supported by the negative correlation between the L/S ratio and the severity of ossification in middle-aged patients. Although few studies have investigated the association between fatty liver and heterotopic ossification, recent research on the gene expression profile of human heterotopic ossification samples suggests its association with NAFLD^35^. Of particular note is that changes in lifestyle and dietary habits over the past three decades have led to a pandemic of obesity and NAFLD among Asians^36,37^. Given that OPLL is particularly common in East Asians^1–4^, NAFLD may become an important risk factor for OPLL progression.

Interestingly, Interestingly, the severity and extent of spinal ligament ossification in the entire spine was correlated with the L/S ratio, rather than the BMI. This indicates that central obesity plays an important role in the progression of heterotopic spinal ossification, and that factors other than mechanical stress are involved in the heterotopic ossification process. Possible candidate factors linking central obesity/fatty liver and OPLL comprise hepatokines and adipokines, as their levels are altered by the accumulation of excessive liver and visceral fat. Insulin-like growth factor 1 (IGF-1) is mainly produced in the liver and acts directly on osteoblasts and osteoclasts to promote bone formation and resorption^38–40^. Goto et al. reported that IGF-1 induced osteogenic differentiation in ligamental-cultured cells from patients with OPLL, compared to that in non-OPLL control cells^41^. Additionally, adipokines, such as leptin, have both cytokine and hormonal properties, and play central and peripheral roles in bone metabolism^42,43^. It was previously reported that serum leptin levels are higher in patients with OPLL than in patients without OPLL^44^. Furthermore, the Zucker fatty rat, which has a missense mutation in the leptin receptor gene, is an animal model of OPLL^45^. The fact that Asians are more prone to central obesity, NAFLD, and type 2 diabetes mellitus than Europeans, partly due to body composition differences in fat and muscle, may also contribute to the higher prevalence of OPLL among Asians^26,46^.

Additionally, the finding that elderly patients with diffuse-OPLL may be at a higher risk of liver fibrosis than those with localized-OPLL supports the idea that fatty liver in middle age contributes to the progression of heterotopic ossification of the spinal ligaments. The fact that OPLL is presumed to develop in patients during their 50 s and 60 s, or at a younger age, may also support this idea^23–25^. An acceptable interpretation for the decreased prevalence of fatty liver in elderly patients with OPLL is that fatty liver progresses to liver fibrosis with age and is no longer diagnosed as fatty liver. It has been reported that fat deposits in hepatocytes tend to disappear as liver fibrosis progresses in patients aged of ≥ 70^20–23^. NAFLD is also important as a comorbidity of OPLL because NAFLD and its pathologically more severe form, non-alcoholic steatohepatitis (NASH), can cause cirrhosis and liver cancer^10,11^. Thus, patients with OPLL with myelopathy can have a significantly impaired quality of life, and their liver condition requires close attention.

This study has several limitations. First, for ethical reasons, we did not perform liver biopsies, which are required to confirm fatty liver or liver fibrosis^18,19^. Therefore, we could not determine the true prevalence of NAFLD, including NAFL and NASH with liver fibrosis, in patients with OPLL. Instead, we evaluated the presence of NASH using the FIB-4 index, which was calculated from standard laboratory tests and age. A FibroScan and/or MR elastography may be useful for diagnosing NASH in future studies. Second, it remains unclear whether NAFLD or NASH is the cause or consequence of heterotopic spinal ligamentous ossification because this study was cross-sectional in nature. Longitudinal or experimental animal model studies are required to reach a conclusion on this topic. Finally, the sample size was small and the statistical power was low because of the rarity of patients with OPLL, especially those with diffuse-OPLL (thoracic OPLL has a prevalence of 0.8%)^6^. Thus, our findings should be validated in multicenter and nationwide studies in the future.

In summary, patients with OPLL had a high prevalence of NAFLD, and the severity of fatty liver was associated with the severity of spinal ligament ossification. Furthermore, elderly patients with diffuse-OPLL may be at risk of advanced liver fibrosis, due to fatty liver disease, compared to those with localized-OPLL. The close association between NAFLD and OPLL demonstrated in this study warrants further study to elucidate the causal nature of this relationship.

## Data Availability

The datasets generated during and/or analyzed during the current study are available from the corresponding author on reasonable request.
